# Quasi-MS^n ^identification of flavanone 7-glycoside isomers in *Da Chengqi Tang *by high performance liquid chromatography-tandem mass spectrometry

**DOI:** 10.1186/1749-8546-4-15

**Published:** 2009-07-24

**Authors:** Fengguo Xu, Ying Liu, Zunjian Zhang, Cheng Yang, Yuan Tian

**Affiliations:** 1 Key Laboratory of Drug Quality Control and Pharmacovigilance (Ministry of Education), China Pharmaceutical University, Nanjing, Jiangsu 210009, PR China; 2 Center for Instrumental Analysis, China Pharmaceutical University, Nanjing, Jiangsu 210009, PR China; 3 Department of Pharmacy, Nanjing Municipal Hospital of Traditional Chinese Medicine, Nanjing, Jiangsu 210001, PR China

## Abstract

**Background:**

*Da Chengqi Tang *(DCT) is a common purgative formula in Chinese medicine. Flavanones are its major active compounds derived from *Fructus Aurantii Immaturus*. The present study developed an LC-MS/MS method to characterize two pairs of flavanone 7-glycoside isomers, i.e., hesperidin versus neohesperidin and naringin versus isonaringin.

**Methods:**

After solid phase purification, components in sample were separated on a Agilent zorbax SB-C18 (5 μm, 250 mm × 4.6 mm) analytical column. ESI-MS and quasi-MS^n ^were performed in negative ion mode to obtain structural data of these two pairs of flavanone 7-glycoside isomers. Moreover, UV absorption was measured.

**Results:**

There was no intra-pairs difference in the UV-Vis and MS/MS spectra of the two pairs of 7-glycoside isomers, whereas the mass spectrometry fragmentation pathways between pairs were different.

**Conclusion:**

The present study developed a LC-MS/MS method to explore the inter- and intra-pair difference of two pairs of flavanone 7-glycoside isomers.

## Background

Described in *Shanghan Lun *(*Treatise on Cold Damage Diseases*, a Chinese medicine classic from the Han dynasty) [1], *Da Chengqi Tang *(DCT) is a well known purgative formula consisting of *Radix et Rhizoma Rhei *(*Dahuang*), *Cortex Magnoliae officinalis *(Houpu), *Fructus Aurantii Immaturus *(*Zhishi*) and *Natrii Sulfas *(*Mangxiao*). DCT is usually used to treat diseases such as acute intestinal obstruction without complications, acute cholecystitis and appendicitis [2]. DCT is also used in treating posttraumatic respiratory distress syndrome [3], reducing acute-phase protein levels in patients with multiple organ failure syndromes [4] and relieving inflammation in patients after tumor operation [5]. Recently DCT was found to possess anti-inflammatory effects apart from its purgative activities [6].

**Figure 1:**
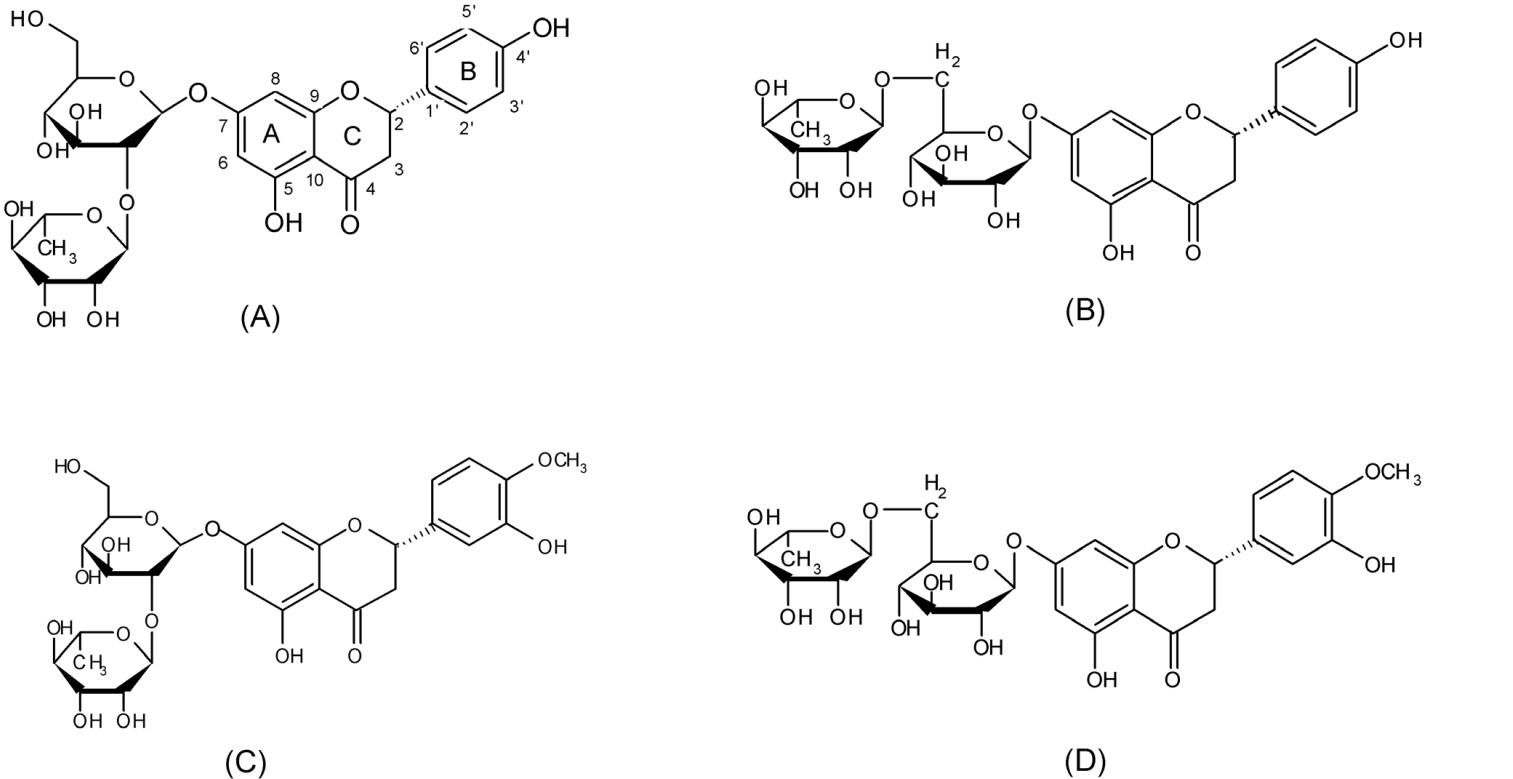
**Structures of naringin (A), isonaringin (B), neohesperidin (C) and hesperidin (D)**.

Flavanones are a major type of active components in DCT [1,6]. Various studies have revealed a variety of pharmacological activities that citrus flavanones possess, such as enzyme inhibition, free radical scavenging, anti-inflammation, anti-estrogen and inhibition of tumor progression [7-13]. Moreover, flavanones have isomers. Our previous study [14] found that hesperidin and neohesperidin, naringin and isonaringin (Figure 1) are two pairs of flavanone 7-glycoside isomers in DCT. The relationships between the chemical structures and their chromatography retention time, ultraviolet-visible (UV-Vis) spectra, MS/MS spectra are yet to be investigated.

HPLC methods, either alone or combined, have been used to determine hesperidin, naringin and neohesperidin in Chinese medicinal materials [15-19]. Meanwhile, flavonoids were also studied via mass spectra fragmentation pathway [20-22]. Zhou et al. [23] described a liquid chromatography-electrospray mass spectrometry (LC-ESI/MS) method to characterize O-diglycosyl flavanones of *Fructus Aurantii *(*Zhiqiao*) and ultra-pressure liquid chromatography (UPLC) retention parameters method to delineate the structure-retention relationship of these O-diglycosyl flavanones. The multiple stage mass spectra fragmentation pathway especially the C-ring related fragmentations of these flavanones are to be studied.

Triple stage quadruple (TSQ) tandem mass spectrometry is used to quantify chemicals and provide sufficient structural information through MS/MS spectra. Kevin *et al. *[24] first reported a quasi-MS^n ^(up to MS^3^) method for analyzing isomeric sulfonamide in milk. The quasi-MS^n ^method is now widely accepted [25,26].

The present study aims to develop a rapid solid-phase extraction LC-MS/MS method to investigate the intra- and inter-pair difference of two pairs of flavanone 7-glycoside isomers in terms of peak retention time, UV-Vis spectra and multistage MS spectra in accordance with the quasi-MS^n ^method.

## Methods

### Chemical and reagents

Reference standards for hesperidin (Batch No: 110721-200512) and naringin (Batch No: 110722-200309) were purchased from the Chinese National Institute for the Control of Pharmaceutical and Biological Products (China). Neohesperidin (Batch No: 05-1010) was purchased from the Shanghai Research and Development Center for Standardization of Chinese Medicines (China). Methanol (HPLC grade) was purchased from VWR International (Germany). Formic acid (analytical grade) was purchased from Nanjing Chemical Reagent First Factory (China). Water was distilled twice before use.

The medicinal herbs and material used in the present study, i.e. *Radix et Rhizoma Rhei, Cortex Magnoliae officinalis, Fructus Aurantii Immaturus *and *Natrii Sulfas*, were purchased from a traditional Chinese medicine shop in Nanjing, China. Prof Ping Li at the Key Laboratory of Modern Chinese Medicines, Pharmaceutical University, China authenticated the medicinal herbs and material using microscopic identification method.

### Instruments and conditions

High-performance liquid chromatography-diode array detection (HPLC-DAD) analysis was performed on a liquid chromatography system (Agilent 1100 Series, Agilent Technologies, USA) equipped with a quadruple pump and a DAD detector. Data were acquired and processed with HP ChemStation software. Chromatography was carried out on an Aglient Zorbax SB-C18 column (5 μm, 250 mm × 4.6 mm) at 45°C column temperature. Mobile phase: methanol/water (0.2% formic acid) = 60/40, flow rate 1.0 ml/min. Peaks were monitored at 285 nm and UV spectra were recorded.

LC-MS/MS experiments were conducted on a Finnigan Surveyor HPLC system (Thermo Electron, USA) coupled with a Finnigan autosampler. The HPLC eluant from the column was introduced into (via a 1:4 split) a Finnigan TSQ Quantum Discovery Max system (Thermo Electron, USA) coupled with an electrospray ionization source. The mass detection was conducted on. The spray voltage was 4.5 kv and the capillary temperature was 300°C. Nitrogen was used as nebulizing and auxiliary gas. The nebulizing gas back-pressure was set at 40 psi and auxiliary gas at 20 psi. Argon was used as the collision gas in MS/MS. Data acquisition was performed with Xcalibur 1.2 software (Thermo Finnigan, USA).

### Improved quasi-MS^n ^approach

We modified the quasi-MS^n ^method [24-26] for the present study. In the first order mass spectrometry, the source collision-induced dissociation (source CID) and CID were set at 5 and 0 respectively to obtain the first order precursor ions. The quasi-MS/MS and MS/MS spectra were obtained under condition set A (source CID = 70, CID = 0) and condition set B (source CID = 5, CID = 30) respectively and were compared to selected quasi-second order precursor ions. The selected quasi-second order precursor ions underwent CID in the collision quadruple to yield useful structure-specific product ions which are referred to as quasi-MS/MS/MS ions. The comparison of the spectra of quasi-MS/MS and of quasi-MS/MS/MS may yield the quasi-third order precursor ions. The quasi-MS^4 ^spectra may be obtained through collision of the ions with argon in CID.

### Preparation of stock solutions

Stock solutions of neohesperidin and naringin were prepared at the concentration of 1.0 mg/ml in methanol and water (45:55) and stored at 4°C. Stock solution of hesperidin was prepared at 0.5 mg/ml in methanol and water (45:55) and stored at 4°C. The stock solutions were further diluted to working solutions at room temperature.

### Preparation of DCT

DCT was prepared in accordance with *Shanghan Lun *[14]. *Cortex Magnoliae officinalis *(24 g) and *Fructus Aurantii Immaturus *(15 g) were immersed in 500 ml distilled water and boiled to 250 ml respectively. The two water extracts were combined. *Radix et Rhizoma Rhei *(12 g) was then immersed in the combined water extracts and boiled to half volume. *Natrii Sulfas *(6 g) was dissolved in the water extract, filtered and diluted to 1000 ml with distilled water and stored at 4°C until use.

### Sample pre-treatments

For quantitative analysis, an aliquot (1.0 ml) of DCT was purified with Lichrospher solid-phase extraction (SPE) cartridge (250 mg packing, Hanbang, China). The cartridge was firstly conditioned with 2 ml of methanol and then equilibrated with 2 ml of water. After sample was loaded, the cartridge was washed with 2.0 ml of methanol (30%). The analyte was eluted with 2.0 ml of methanol (45%) and diluted with 50% methanol to a final volume of 5 ml. The solution was filtered through a syringe organic membrane filter (0.45 μm, Hanbang, China) before HPLC analysis.

## Results and discussion

### Optimization of sample pre-treatments procedure and separation conditions

Various methods including liquid-liquid extraction and solid-phase extraction were tested during method development and sample pre-treatment with C_18 _cartridges was adopted. A solution of methanol (30%) in water was optimized to wash the co-existing hydrophilic components after sample was loaded. A solution of methanol (45%) in water was used to elute and separate the four target analytes from the lipophilic components.

**Figure 2:**
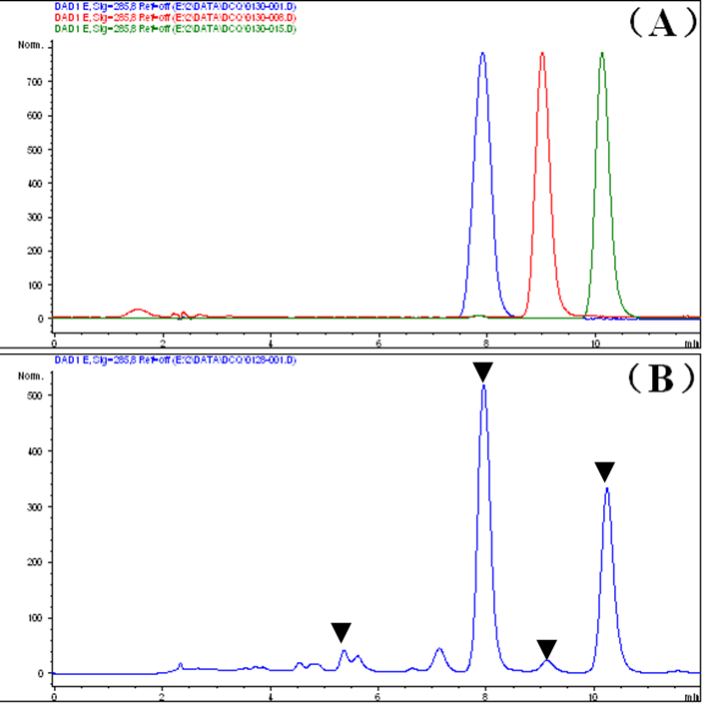
**Representative HPLC/UV chromatograms of reference standards (A) and DCT (B)**. The retention times of isonarigin, naringin, hesperetin and neohesperetin were 5.5, 8.0, 9.3 and 10.3 minutes respectively (marked with black triangle).

**Figure 3:**
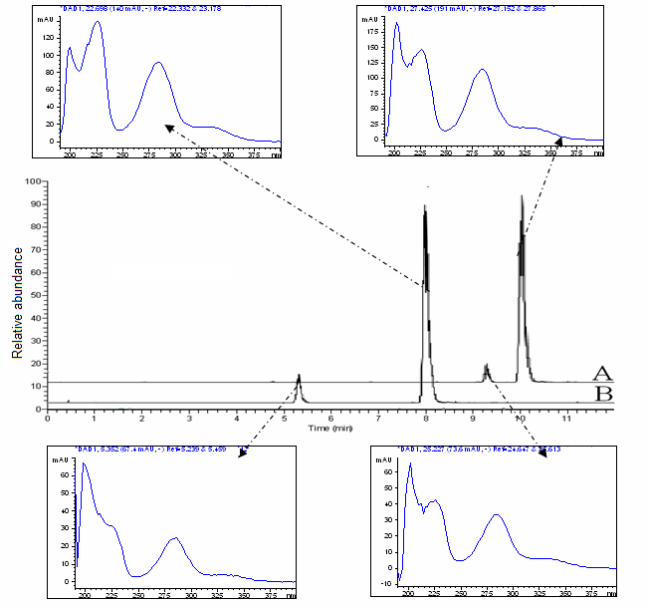
**Extracted ions chromatogram (EIC) of *m/z *609.00 (A) and *m/z *579.00 (B) combined with the UV spectra**. The retention times of isonarngin, naringin, hesperetin and neohesperetin were 5.5, 8.0, 9.3 and 10.3 minutes respectively.

After testing several mobile phases systematically, we reached the chromatographic conditions used in the present study. The addition of formic acid to the solvent system, i.e. methanol and water with 0.2% formic acid (60:40), and the column temperature (45°C) improved the separation of naringin, hesperetin and neohesperetin and helped obtain symmetrical and sharp peaks. The retention times of naringin, hesperetin and neohesperetin were 8.0, 9.3 and 10.3 minutes respectively (Figures 2 and 3).

### Identification of the two pairs of flavanone 7-glycoside isomers by LC-MS/MS

We found that hesperidin, neohesperidin and naringin corresponded to the peaks at retention times of 9.3, 10.3 and 8.0 minutes respectively. Neohesperidin and naringin were of the same neohesperidose at the carbon 7 of the A-ring and that hesperidin and isonaringin had rutinose at the same position. Meanwhile, the chromatographic retention times of hesperidin and neohesperidin suggested that the same parent chemical structure with rutinose would contribute less than that with neohesperidose under the same chromatographic conditions. Therefore we predicted that the retention time of isonaringin would be less than that of naringin.

An extracted ions chromatography (EIC) of *m/z *579.00 and *m/z *609.00 to demonstrated that the peaks at retention times of 9.3 and 10.3 minutes were hesperidin and neohesperidin respectively (Figure 3A) and that the peaks at retention times of 5.5 and 8.0 minutes were isonaringin and naringin respectively (Figure 3B).

### Mass spectrometric structure characterization of naringin and isonaringin

**Figure 4:**
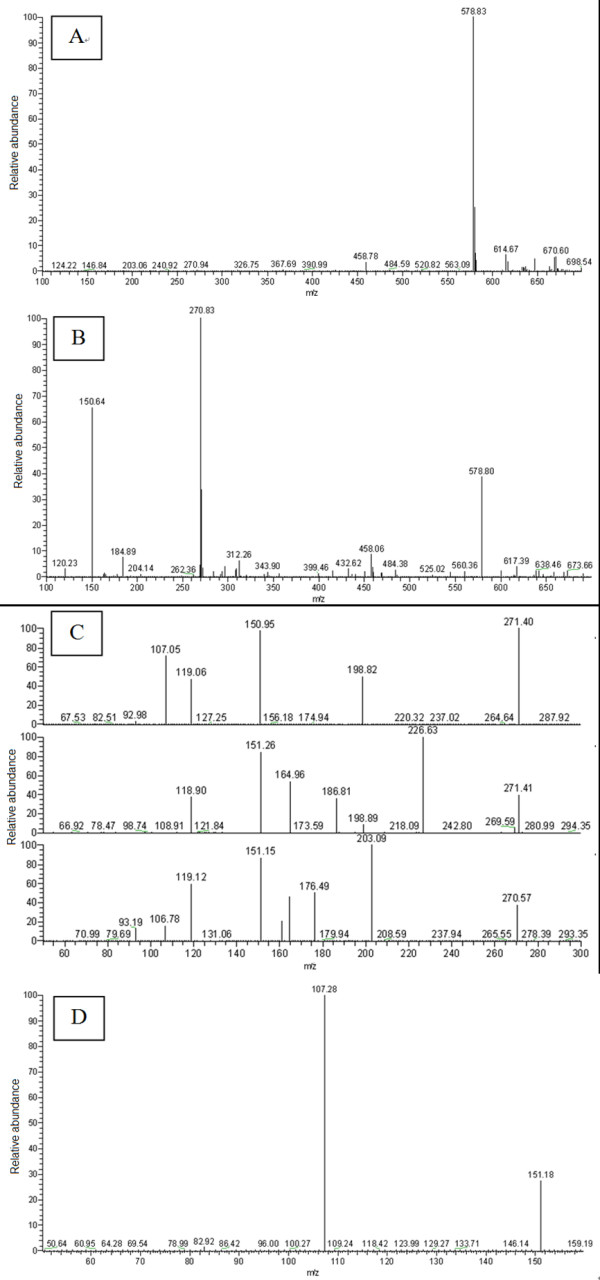
**Representative first order mass spectra of naringin and isonaringin **(A: source CID = 5, CID = 0); Representative quasi-MS/MS from first order precursor ions *m/z *579.00 for naringin and isonaringin (B: source CID = 70, CID = 0); Representative quasi-MS/MS/MS from quasi-second order precursor ions *m/z *271.40 for naringin and isonaringin (C: source CID = 70, CID = 30);. Representative quasi-MS^4 ^from quasi-third order precursor ions *m/z *151.16 for naringin and isonaringin (D: source CID = 70, CID = 30)

From the first order mass spectrometry scan spectra (source CID = 5 v, CID = 0 v) of naringin and isonaringin (Figure 4A), we found that the [M-H]^- ^ions were both at *m/z *579. Ions of *m/z *271 and *m/z *151 were the major product ions of [M-H]^- ^*m/z *579 under the condition set A (quasi-MS/MS spectra: source CID = 70 v, CID = 0 v) and condition set B (MS/MS spectra: source CID = 5 v, CID = 30 v) (Figure 4B). After comparing the two spectra, we selected *m/z *271 as quasi-second order precursor ions which underwent CID = 30 v to obtain quasi-MS/MS/MS spectra. We found an unusual phenomenon in the quasi-MS/MS/MS spectra from *m/z *271, i.e. the ions of *m/z *227, 203, 199,187, 176, 165, 161 and 107 did not always appear at each time point of the peaks in total ions chromatography (TIC) for naringin and isonaringin, while ions at *m/z *151 and 119 were found at each time point of the peaks in TIC for the two isomers (Figure 4C). This phenomenon suggested that there might be many unstable parallel fragmentation pathways from *m/z *271. We isolated the quasi-third order precursor ions of *m/z *151 by comparing the spectra of quasi-MS/MS (from [M-H]^- ^*m/z *579, source CID = 70 v, CID = 0 v) and quasi-MS/MS/MS (from quasi-second order precursor ion *m/z *271, source CID = 70 v, CID = 30 v) and then impacted in CID with collision gas (argon; source CID = 70 v; CID = 30 v). We obtained the quasi-MS^4 ^spectra (Figure 4D) from *m/z *151, through which we confirmed that ions *m/z *107 were generated from *m/z *151.

**Figure 5:**
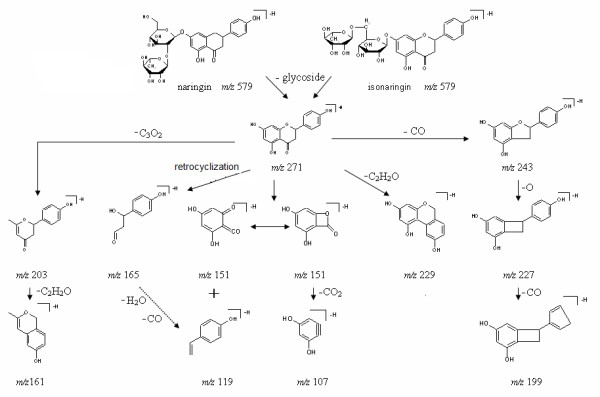
**Proposed fragmentation of naringin and isonaringin based on quasi-MS^n ^(up to MS^4^) spectra in negative ion mode**.

Figure 5 shows the proposed fragmentation pathway of naringin and isonaringin based on the quasi-MS^n ^method. The quasi-second order precursor ions at *m/z *271 were generated from [M-H]^- ^*m/z *579 after the neutral loss of glycoside: rutinose and neohesperidose for isonaringin and naringin respectively. The most dominate ions of *m/z*151 and 119 from *m/z *271 were yielded through retro-Diels-Alder (RDA) reactions by breaking two C-C bonds of C-ring, which gave structurally informative ions of A-ring and B-ring. Concerning the RDA fragmentations, we noted that the ions at *m/z *151 underwent further CO_2 _loss leading to a fragment at *m/z *107 [21], which appeared both in quasi-MS/MS/MS spectra from *m/z *271 and quasi-MS^4 ^spectra from *m/z *151. The ion at *m/z *203 was proposed to be generated from *m/z *271 through the C_3_O_2 _neutral loss and underwent further C_2_H_2_O loss to fragment at *m/z *161, which occurred mainly on the C-ring followed by a new cyclization implying the B-ring. In general, all flavones exhibited neutral losses of CO and CO_2 _that may be attributed to the C-ring [21]. Therefore the ions at *m/z *243 and 227 were proposed as products of the losses of CO and CO_2 _respectively. The ions at *m/z *227 would undergo further loss of CO at the position carbon 4' of B-ring and generate ion at *m/z *199. A retrocyclisation fragmentation involving the 0 and 4 bonds was proposed to form fragments *m/z *165 from *m/z *271. Therefore another source of *m/z *119 was proposed as a result of the loss CO and H_2_O from *m/z *165.

### Mass spectrometric structure characterization of hesperidin and neohesperidin

**Figure 6:**
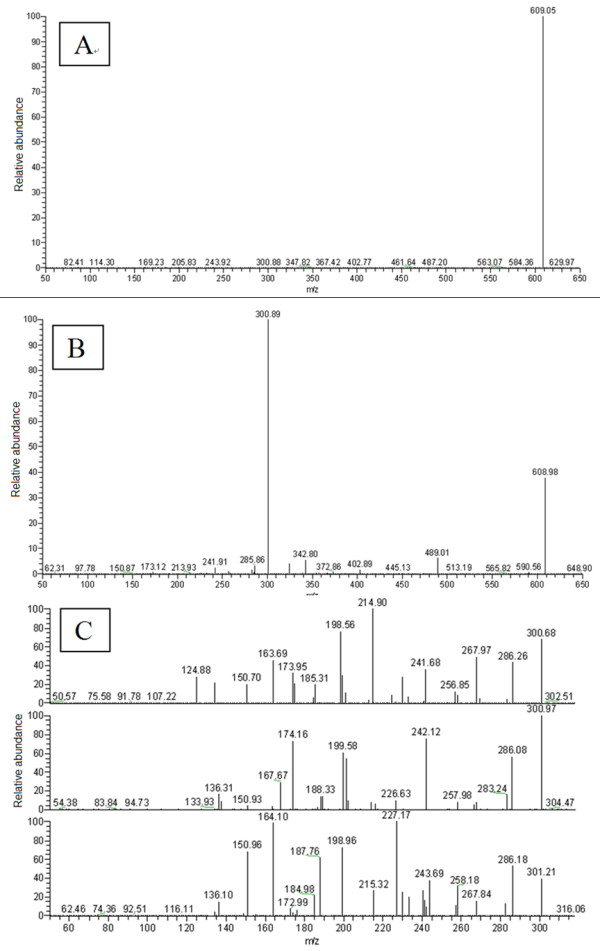
**Representative first order mass spectra of hesperidin and neohesperidin **(A: source CID = 5, CID = 0); Representative quasi-MS/MS from first order precursor ions *m/z *609.00 for hesperidin and neohesperidin (B: source CID = 70, CID = 0); Representative quasi-MS/MS/MS from quasi-second order precursor ions for hesperidin and and neohesperidin (C: source CID = 70, CID = 30)

The MS spectra of hesperidin and neohesperidin obtained by quasi-MS^n ^(up to MS^3 ^for this pair of flavanone 7-glycoside isomer) method are shown in Figure 6. The [M-H]^- ^ions of hesperidin and neohesperidin obtained from the first order mass spectrometry scan spectra (source CID = 5 v; CID = 0 v) were both at *m/z *609. Ions of *m/z *301 were the major product ions of [M-H]^- ^under the condition set A (quasi-MS/MS spectra: source CID = 70 v, CID = 0 v) and condition set B (MS/MS spectra: source CID = 5 v, CID = 30 v). Ions at *m/z *301 were selected as quasi-second order precursor ions and underwent CID = 30 v to obtain quasi-MS/MS/MS spectra. Here we found the same unusual phenomenon, i.e. the ions at *m/z *268, 257, 241, 227, 215, 174, 168, 164, 151, 136 and 125 were not always at each time point of the peaks in TIC for hesperidin and neohesperidin except the ions at *m/z *286. This phenomenon suggested that there might be many unstable parallel fragmentation pathways from *m/z *301.

**Figure 7:**
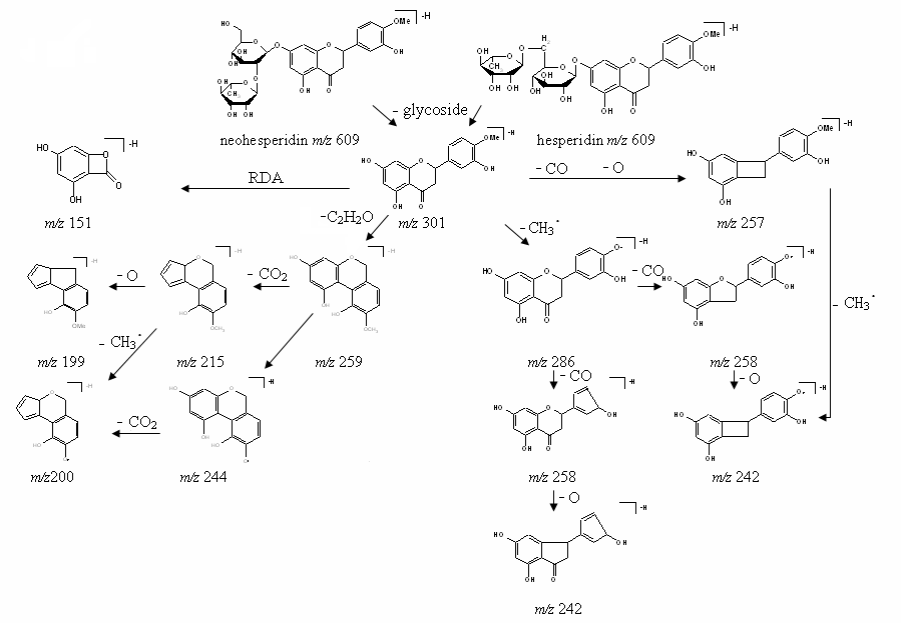
**Proposed fragmentation of neohesperidin and hesperidin based on quasi-MS^n ^(up to MS^3^) spectra in negative ion mode**.

Figure 7 shows the proposed fragmentation pathway of hesperidin and neohesperidin. The mass spectrometry fragmentation behaviors of hesperidin and neohesperidin were different from those of naringin and isonaringin. The most stable ions at *m/z *286 were not generated through RDA reactions but formed after the CH_3_· loss from *m/z *301. Meanwhile the ions *m/z *151 yielded from *m/z *301 by breaking two C-C bonds of C-ring became weak and were not in the quasi-MS/MS spectra from *m/z *609. The ion at *m/z *259 was generated from *m/z *301 through the C_3_O_2 _neutral loss. A further loss of CO_2 _and CH_3_· from these ions led to fragments at *m/z *215 and 244 respectively. After further O loss from C-ring, *m/z *199 were formed from *m/z *215. Ions at *m/z *200 were generated through two pathways: one was the CH_3_· loss from ions at *m/z *215 and the other was the CO_2 _loss at A-ring from ions at *m/z *244. Two ions generated through CO loss from *m/z *286 were attributed to the abundance of fragments at *m/z *258. One of the two ions was generated at carbon 4' of B-ring while the other was formed involved carbon 4 of C-ring. The two ions formula of *m/z *258 both underwent further O loss from C-ring and generated two ions formula of *m/z *242.

### Fragmentation pathways comparison of the two pairs of flavanone 7-glycoside isomers: hesperidin versus neohesperidin and naringin versus isonaringin

We found that the mass spectrometry fragmentation behaviors between the two pairs of flavanone 7-glycoside isomers were very different, while the within pair mass spectrometry behaviors were very similar.

For hesperidin and neohesperidin, the fragment ions were related to CH_3_· loss at the position of carbon 4' of B-ring. It indicated that the bond between O and CH_3 _was found at an active fragmentation position. The very common fragmentation by retro-Diels-Alder (RDA) reactions in terms of flavonoid aglycone became inactive for hesperidin and neohesperidin compared with naringin and isonaringin. The reason for this was possibly that there was no active fragmentation bond between O and CH_3 _in A-ring and B-ring for naringin and isonaringin. Therefore the RDA reactions became the dominate fragmentation pathway for naringin and isonaringin.

## Conclusion

The present study developed a LC-MS/MS method to explore the inter- and intra-pairs difference of two pairs of flavanone 7-glycoside isomers: hesperidin versus neohesperidin and naringin versus isonaringin in DCT in regards to peak retention time, UV-Vis spectra, multistage MS spectra obtain by quasi-MS^n ^(up to MS^4^) method.

## Abbreviations

CID: collision-induced dissociation; DCT: *Da Chengqi Tang*; EIC: extracted ions chromatogram; ESI-MS: electrospray mass spectrometry; LC-ESI/MS: liquid chromatography-electrospray mass spectrometry; HPLC-DAD: high-performance liquid chromatography-diode array detection; MS/MS: tandem mass spectrometry; RDA: retro-Diels-Alder; SPE: solid-phase extraction; TIC: total ions chromatogram; TSQ: triple stage quadrupole; UPLC: ultra-pressure liquid chromatography; UV-Vis: ultraviolet-visible

## Competing interests

The authors declare that they have no competing interests.

## Authors' contributions

FGX and YL performed the instrumental experiments and drafted the manuscript. CY and YT analyzed the data and revised the manuscript. ZJZ supervised all the experiments and revised the manuscript. All authors read and approved the final version of the manuscript.
